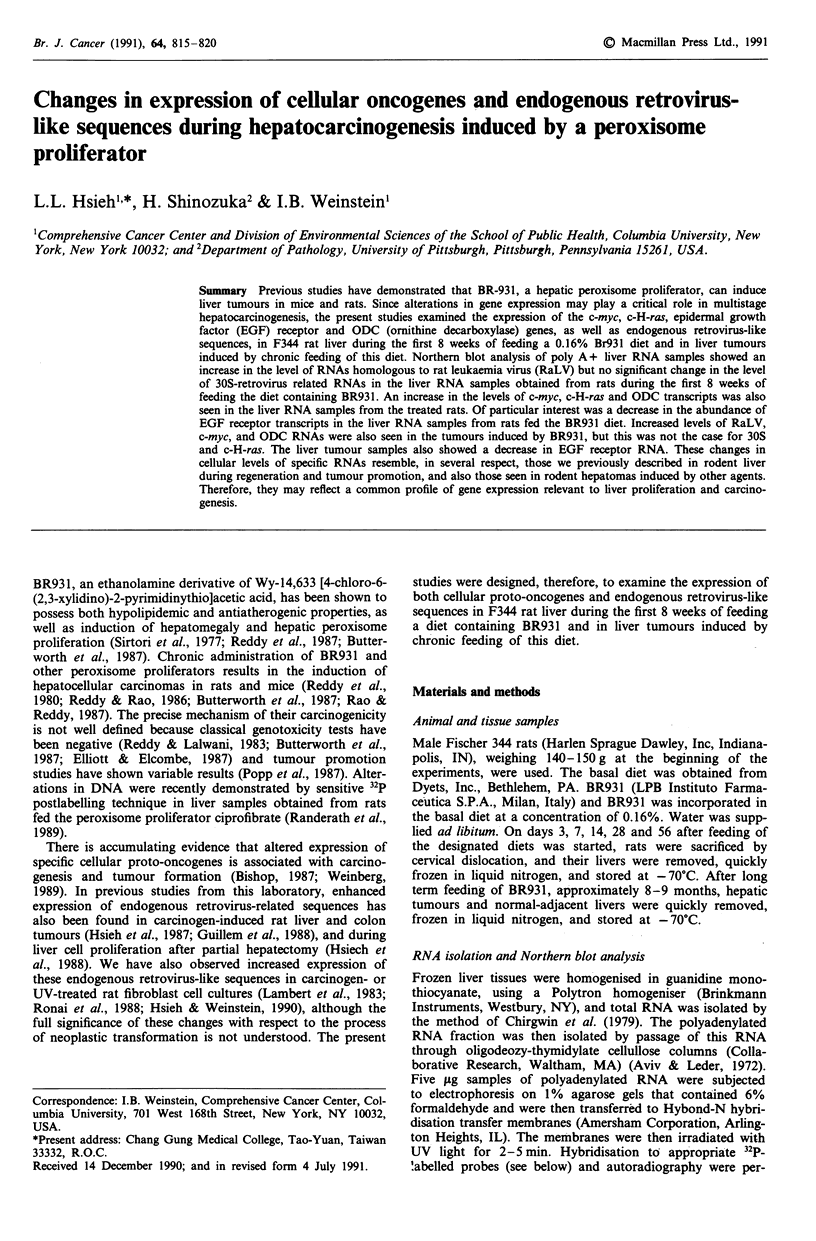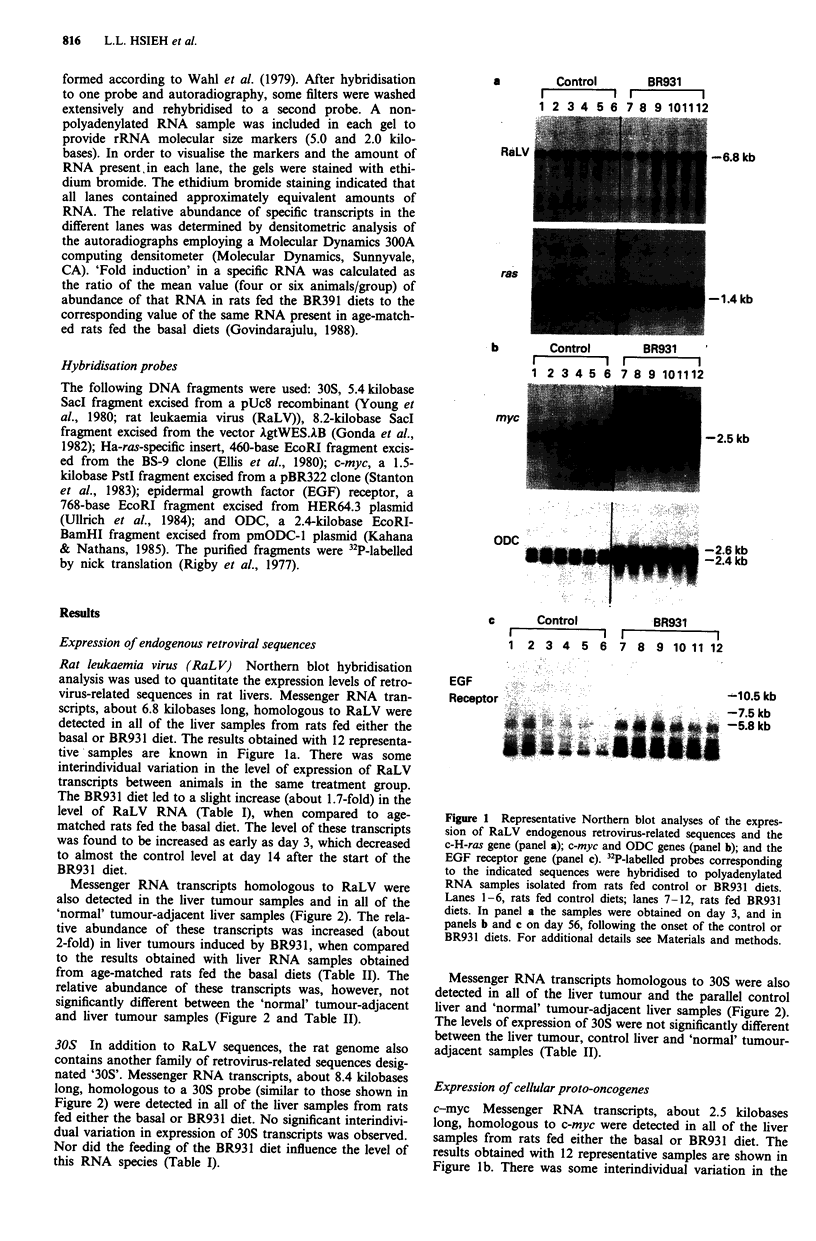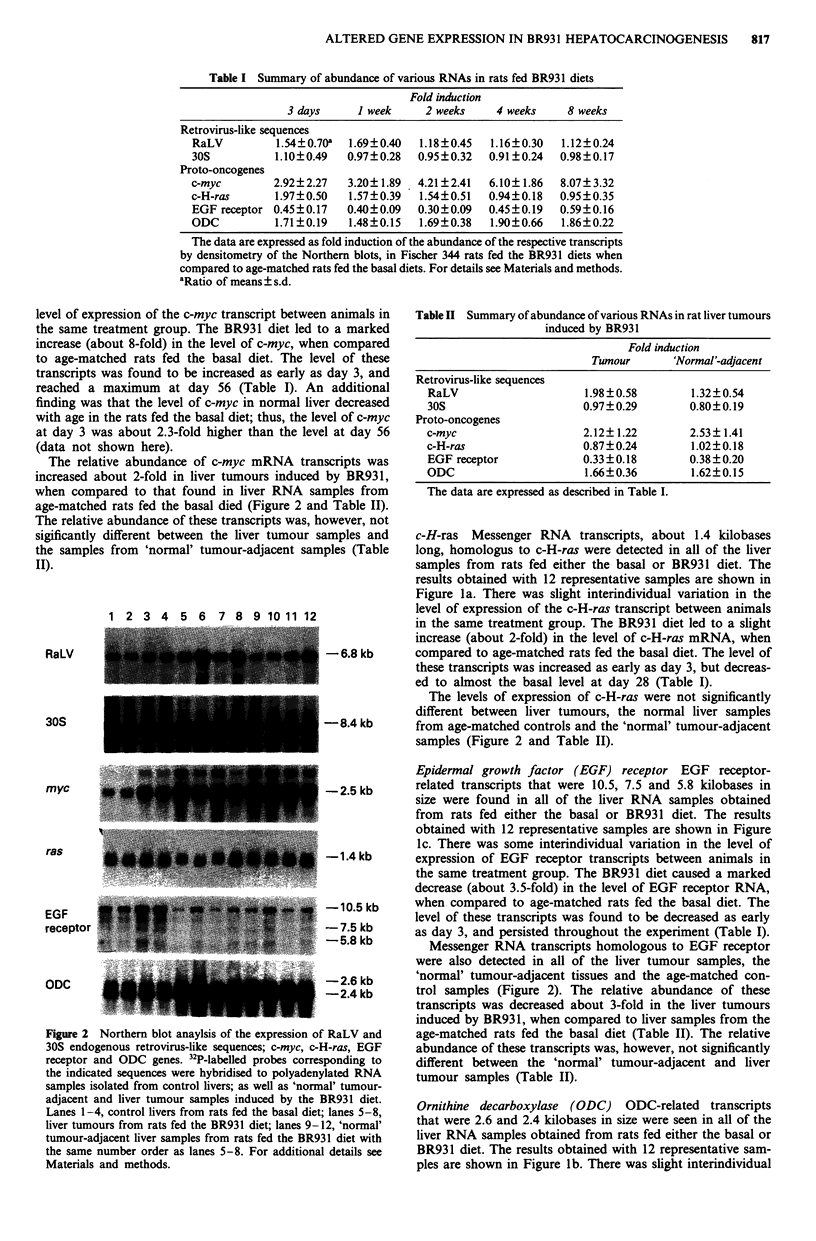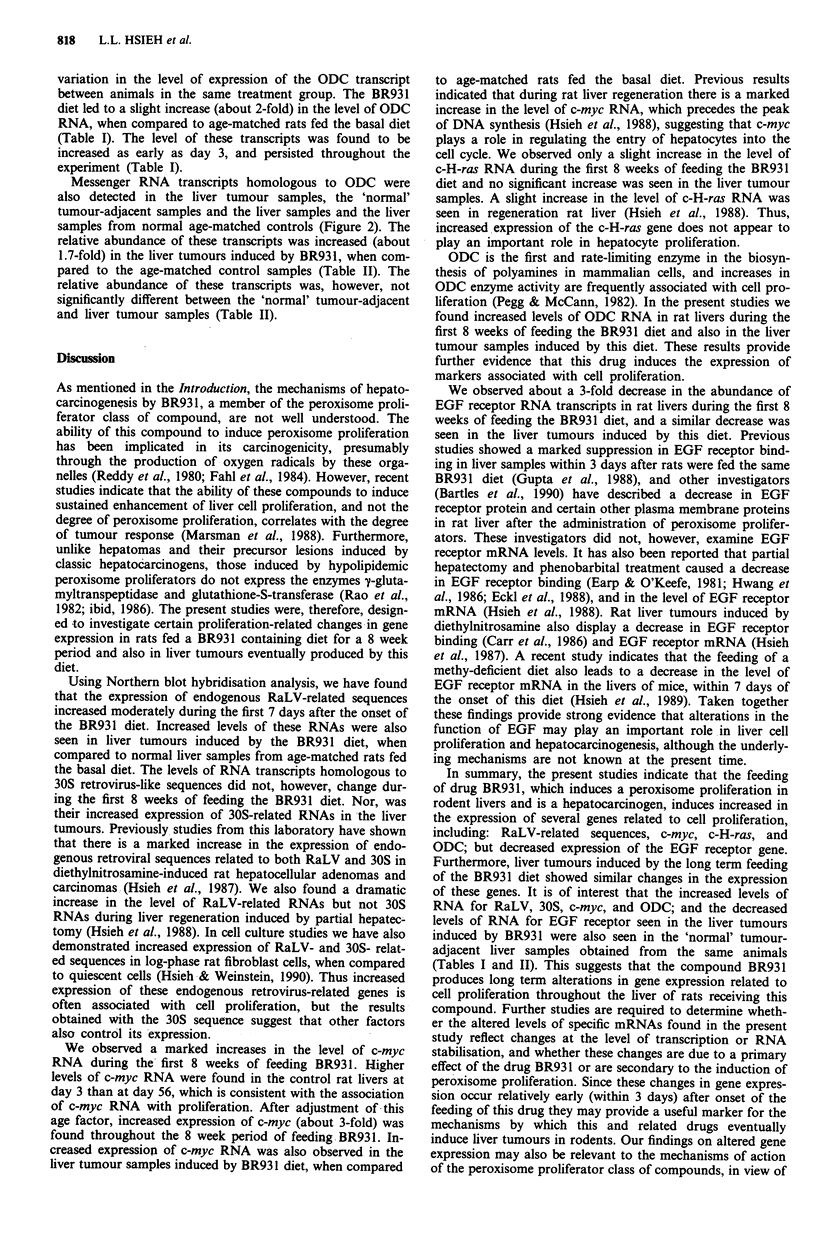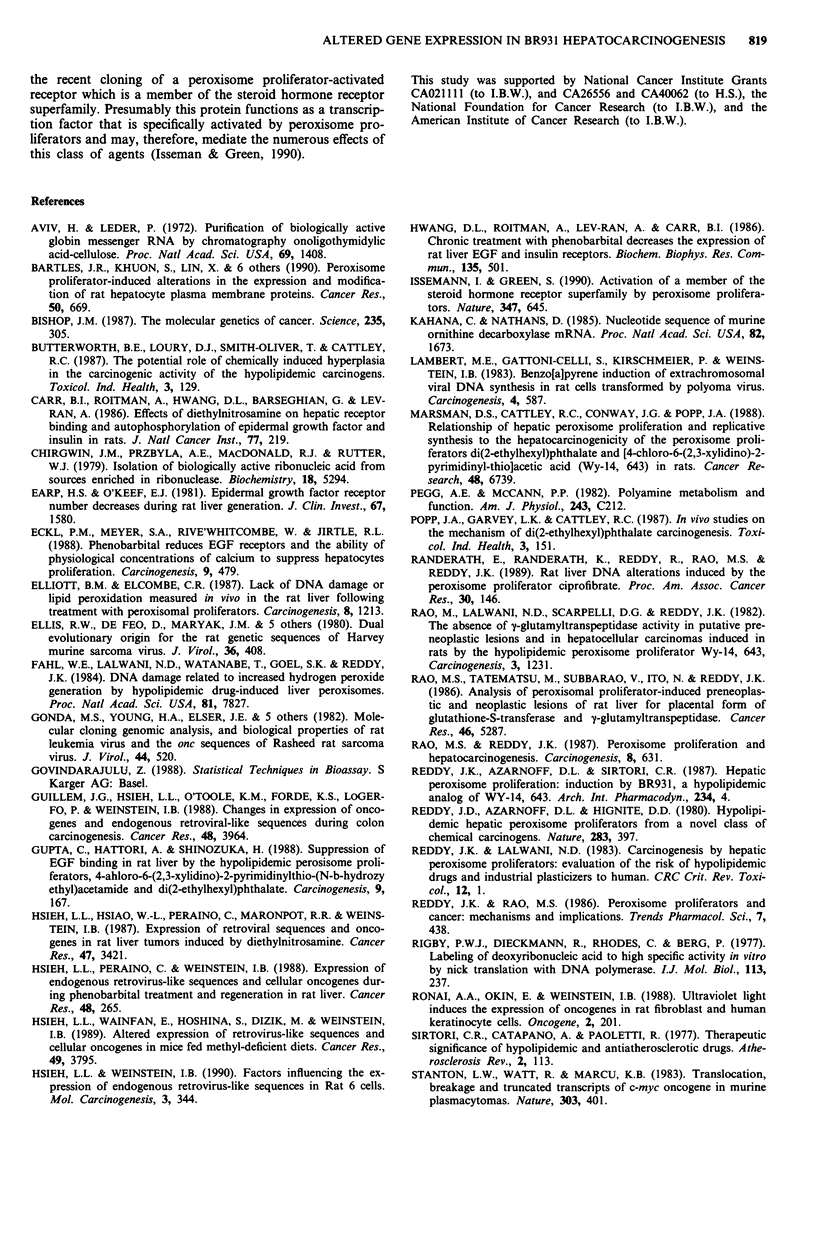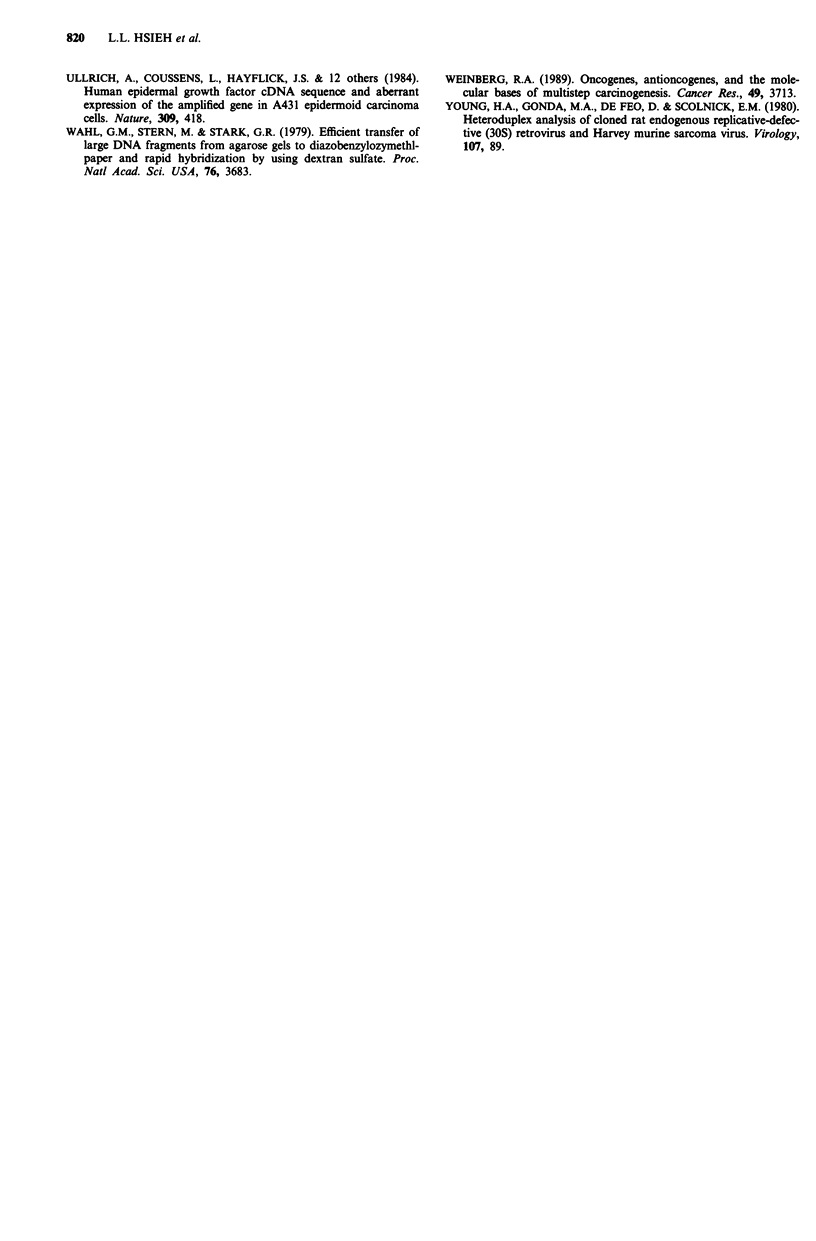# Changes in expression of cellular oncogenes and endogenous retrovirus-like sequences during hepatocarcinogenesis induced by a peroxisome proliferator.

**DOI:** 10.1038/bjc.1991.406

**Published:** 1991-11

**Authors:** L. L. Hsieh, H. Shinozuka, I. B. Weinstein

**Affiliations:** Comprehensive Cancer Center, School of Public Health, Columbia University, New York, New York 10032.

## Abstract

**Images:**


Br. J. Cancer (1991), 64, 815-820 © Macmillan Press Ltd., 1991

### Changes in expression of cellular oncogenes and endogenous retrovirus-like sequences during hepatocarcinogenesis induced by a peroxisome proliferator

L.L. Hsieh[1,*], H. Shinozuka[2] & I.B. Weinstein[1]

[1]*Comprehensive Cancer Center and Division of Environmental Sciences of the School of Public Health, Columbia University, New York, New York 10032; and* [2]*Department of Pathology, University of Pittsburgh, Pittsburgh, Pennsylvania 15261, USA.*

**Summary** Previous studies have demonstrated that BR-931, a hepatic peroxisome proliferator, can induce liver tumours in mice and rats. Since alterations in gene expression may play a critical role in multistage hepatocarcinogenesis, the present studies examined the expression of the c-*myc*, c-H-*ras*, epidermal growth factor (EGF) receptor and ODC (ornithine decarboxylase) genes, as well as endogenous retrovirus-like sequences, in F344 rat liver during the first 8 weeks of feeding a 0.16% Br931 diet and in liver tumours induced by chronic feeding of this diet. Northern blot analysis of poly A+ liver RNA samples showed an increase in the level of RNAs homologous to rat leukaemia virus (RaLV) but no significant change in the level of 30S-retrovirus related RNAs in the liver RNA samples obtained from rats during the first 8 weeks of feeding the diet containing BR931. An increase in the levels of c-*myc*, c-H-*ras* and ODC transcripts was also seen in the liver RNA samples from the treated rats. Of particular interest was a decrease in the abundance of EGF receptor transcripts in the liver RNA samples from rats fed the BR931 diet. Increased levels of RaLV, c-*myc*, and ODC RNAs were also seen in the tumours induced by BR931, but this was not the case for 30S and c-H-*ras*. The liver tumour samples also showed a decrease in EGF receptor RNA. These changes in cellular levels of specific RNAs resemble, in several respect, those we previously described in rodent liver during regeneration and tumour promotion, and also those seen in rodent hepatomas induced by other agents. Therefore, they may reflect a common profile of gene expression relevant to liver proliferation and carcinogenesis.

BR931, an ethanolamine derivative of Wy-14,633 [4-chloro-6-(2,3-xylidino)-2-pyrimidinythio]acetic acid, has been shown to possess both hypolipidemic and antiatherogenic properties, as well as induction of hepatomegaly and hepatic peroxisome proliferation (Sirtori *et al.*, 1977; Reddy *et al.*, 1987; Butterworth *et al.*, 1987). Chronic administration of BR931 and other peroxisome proliferators results in the induction of hepatocellular carcinomas in rats and mice (Reddy *et al.*, 1980; Reddy & Rao, 1986; Butterworth *et al.*, 1987; Rao & Reddy, 1987). The precise mechanism of their carcinogenicity is not well defined because classical genotoxicity tests have been negative (Reddy & Lalwani, 1983; Butterworth *et al.*, 1987; Elliott & Elcombe, 1987) and tumour promotion studies have shown variable results (Popp *et al.*, 1987). Alterations in DNA were recently demonstrated by sensitive $^{32}P$ postlabelling technique in liver samples obtained from rats fed the peroxisome proliferator ciprofibrate (Randerath *et al.*, 1989).

There is accumulating evidence that altered expression of specific cellular proto-oncogenes is associated with carcinogenesis and tumour formation (Bishop, 1987; Weinberg, 1989). In previous studies from this laboratory, enhanced expression of endogenous retrovirus-related sequences has also been found in carcinogen-induced rat liver and colon tumours (Hsieh *et al.*, 1987; Guillem *et al.*, 1988), and during liver cell proliferation after partial hepatectomy (Hsiech *et al.*, 1988). We have also observed increased expression of these endogenous retrovirus-like sequences in carcinogen- or UV-treated rat fibroblast cell cultures (Lambert *et al.*, 1983; Ronai *et al.*, 1988; Hsieh & Weinstein, 1990), although the full significance of these changes with respect to the process of neoplastic transformation is not understood. The present studies were designed, therefore, to examine the expression of both cellular proto-oncogenes and endogenous retrovirus-like sequences in F344 rat liver during the first 8 weeks of feeding a diet containing BR931 and in liver tumours induced by chronic feeding of this diet.

### Materials and methods

### *Animal and tissue samples*

Male Fischer 344 rats (Harlen Sprague Dawley, Inc, Indianapolis, IN), weighing 140-150 g at the beginning of the experiments, were used. The basal diet was obtained from Dyets, Inc., Bethlehem, PA. BR931 (LPB Instituto Farmaceutica S.P.A., Milan, Italy) and BR931 was incorporated in the basal diet at a concentration of 0.16%. Water was supplied *ad libitum*. On days 3, 7, 14, 28 and 56 after feeding of the designated diets was started, rats were sacrificed by cervical dislocation, and their livers were removed, quickly frozen in liquid nitrogen, and stored at −70°C. After long term feeding of BR931, approximately 8-9 months, hepatic tumours and normal-adjacent livers were quickly removed, frozen in liquid nitrogen, and stored at −70°C.

### *RNA isolation and Northern blot analysis*

Frozen liver tissues were homogenised in guanidine monothiocyanate, using a Polytron homogeniser (Brinkmann Instruments, Westbury, NY), and total RNA was isolated by the method of Chirgwin *et al.* (1979). The polyadenylated RNA fraction was then isolated by passage of this RNA through oligodeozy-thymidylate cellullose columns (Collaborative Research, Waltham, MA) (Aviv & Leder, 1972). Five μg samples of polyadenylated RNA were subjected to electrophoresis on 1% agarose gels that contained 6% formaldehyde and were then transferred to Hybond-N hybridisation transfer membranes (Amersham Corporation, Arlington Heights, IL). The membranes were then irradiated with UV light for 2-5 min. Hybridisation to appropriate $^{32}P$-labelled probes (see below) and autoradiography were per-

Correspondence: I.B. Weinstein, Comprehensive Cancer Center, Columbia University, 701 West 168th Street, New York, NY 10032, USA.
*Present address: Chang Gung Medical College, Tao-Yuan, Taiwan 33332, R.O.C.
Received 14 December 1990; and in revised form 4 July 1991.

formed according to Wahl *et al.* (1979). After hybridisation to one probe and autoradiography, some filters were washed extensively and rehybridised to a second probe. A non-polyadenylated RNA sample was included in each gel to provide rRNA molecular size markers (5.0 and 2.0 kilobases). In order to visualise the markers and the amount of RNA present,in each lane, the gels were stained with ethidium bromide. The ethidium bromide staining indicated that all lanes contained approximately equivalent amounts of RNA. The relative abundance of specific transcripts in the different lanes was determined by densitometric analysis of the autoradiographs employing a Molecular Dynamics 300A computing densitometer (Molecular Dynamics, Sunnyvale, CA). 'Fold induction' in a specific RNA was calculated as the ratio of the mean value (four or six animals/group) of abundance of that RNA in rats fed the BR391 diets to the corresponding value of the same RNA present in age-matched rats fed the basal diets (Govindarajulu, 1988).

*Hybridisation probes*

The following DNA fragments were used: 30S, 5.4 kilobase SacI fragment excised from a pUc8 recombinant (Young *et al.*, 1980; rat leukaemia virus (RaLV)), 8.2-kilobase SacI fragment excised from the vector λgtWES.λB (Gonda *et al.*, 1982); Ha-*ras*-specific insert, 460-base EcoRI fragment excised from the BS-9 clone (Ellis *et al.*, 1980); c-*myc*, a 1.5-kilobase PstI fragment excised from a pBR322 clone (Stanton *et al.*, 1983); epidermal growth factor (EGF) receptor, a 768-base EcoRI fragment excised from HER64.3 plasmid (Ullrich *et al.*, 1984); and ODC, a 2.4-kilobase EcoRI-BamHI fragment excised from pmODC-1 plasmid (Kahana & Nathans, 1985). The purified fragments were $^{32}$P-labelled by nick translation (Rigby *et al.*, 1977).

### Results

*Expression of endogenous retroviral sequences*

*Rat leukaemia virus (RaLV)* Northern blot hybridisation analysis was used to quantitate the expression levels of retrovirus-related sequences in rat livers. Messenger RNA transcripts, about 6.8 kilobases long, homologous to RaLV were detected in all of the liver samples from rats fed either the basal or BR931 diet. The results obtained with 12 representative samples are known in Figure 1a. There was some interindividual variation in the level of expression of RaLV transcripts between animals in the same treatment group. The BR931 diet led to a slight increase (about 1.7-fold) in the level of RaLV RNA (Table I), when compared to age-matched rats fed the basal diet. The level of these transcripts was found to be increased as early as day 3, which decreased to almost the control level at day 14 after the start of the BR931 diet.

Messenger RNA transcripts homologous to RaLV were also detected in the liver tumour samples and in all of the 'normal' tumour-adjacent liver samples (Figure 2). The relative abundance of these transcripts was increased (about 2-fold) in liver tumours induced by BR931, when compared to the results obtained with liver RNA samples obtained from age-matched rats fed the basal diets (Table II). The relative abundance of these transcripts was, however, not significantly different between the 'normal' tumour-adjacent and liver tumour samples (Figure 2 and Table II).

*30S* In addition to RaLV sequences, the rat genome also contains another family of retrovirus-related sequences designated '30S'. Messenger RNA transcripts, about 8.4 kilobases long, homologous to a 30S probe (similar to those shown in Figure 2) were detected in all of the liver samples from rats fed either the basal or BR931 diet. No significant interindividual variation in expression of 30S transcripts was observed. Nor did the feeding of the BR931 diet influence the level of this RNA species (Table I).

**Figure 1** Representative Northern blot analyses of the expression of RaLV endogenous retrovirus-related sequences and the c-H-*ras* gene (panel **a**); c-*myc* and ODC genes (panel **b**); and the EGF receptor gene (panel **c**). $^{32}$P-labelled probes corresponding to the indicated sequences were hybridised to polyadenylated RNA samples isolated from rats fed control or BR931 diets. Lanes 1–6, rats fed control diets; lanes 7–12, rats fed BR931 diets. In panel **a** the samples were obtained on day 3, and in panels **b** and **c** on day 56, following the onset of the control or BR931 diets. For additional details see Materials and methods.

Messenger RNA transcripts homologous to 30S were also detected in all of the liver tumour and the parallel control liver and 'normal' tumour-adjacent liver samples (Figure 2). The levels of expression of 30S were not significantly different between the liver tumour, control liver and 'normal' tumour-adjacent samples (Table II).

*Expression of cellular proto-oncogenes*

*c–myc* Messenger RNA transcripts, about 2.5 kilobases long, homologous to c-*myc* were detected in all of the liver samples from rats fed either the basal or BR931 diet. The results obtained with 12 representative samples are shown in Figure 1b. There was some interindividual variation in the

**Table I** Summary of abundance of various RNAs in rats fed BR931 diets

| | Fold induction | | | | |
|---|---|---|---|---|---|
| | *3 days* | *1 week* | *2 weeks* | *4 weeks* | *8 weeks* |
| Retrovirus-like sequences | | | | | |
| RaLV | 1.54±0.70[a] | 1.69±0.40 | 1.18±0.45 | 1.16±0.30 | 1.12±0.24 |
| 30S | 1.10±0.49 | 0.97±0.28 | 0.95±0.32 | 0.91±0.24 | 0.98±0.17 |
| Proto-oncogenes | | | | | |
| c-*myc* | 2.92±2.27 | 3.20±1.89 | 4.21±2.41 | 6.10±1.86 | 8.07±3.32 |
| c-H-*ras* | 1.97±0.50 | 1.57±0.39 | 1.54±0.51 | 0.94±0.18 | 0.95±0.35 |
| EGF receptor | 0.45±0.17 | 0.40±0.09 | 0.30±0.09 | 0.45±0.19 | 0.59±0.16 |
| ODC | 1.71±0.19 | 1.48±0.15 | 1.69±0.38 | 1.90±0.66 | 1.86±0.22 |

The data are expressed as fold induction of the abundance of the respective transcripts by densitometry of the Northern blots, in Fischer 344 rats fed the BR931 diets when compared to age-matched rats fed the basal diets. For details see Materials and methods. [a]Ratio of means±s.d.

level of expression of the c-*myc* transcript between animals in the same treatment group. The BR931 diet led to a marked increase (about 8-fold) in the level of c-*myc*, when compared to age-matched rats fed the basal diet. The level of these transcripts was found to be increased as early as day 3, and reached a maximum at day 56 (Table I). An additional finding was that the level of c-*myc* in normal liver decreased with age in the rats fed the basal diet; thus, the level of c-*myc* at day 3 was about 2.3-fold higher than the level at day 56 (data not shown here).

The relative abundance of c-*myc* mRNA transcripts was increased about 2-fold in liver tumours induced by BR931, when compared to that found in liver RNA samples from age-matched rats fed the basal died (Figure 2 and Table II). The relative abundance of these transcripts was, however, not sigificantly different between the liver tumour samples and the samples from 'normal' tumour-adjacent samples (Table II).

**Figure 2** Northern blot anaylsis of the expression of RaLV and 30S endogenous retrovirus-like sequences; c-*myc*, c-H-*ras*, EGF receptor and ODC genes. $^{32}$P-labelled probes corresponding to the indicated sequences were hybridised to polyadenylated RNA samples isolated from control livers; as well as 'normal' tumour-adjacent and liver tumour samples induced by the BR931 diet. Lanes 1–4, control livers from rats fed the basal diet; lanes 5–8, liver tumours from rats fed the BR931 diet; lanes 9–12, 'normal' tumour-adjacent liver samples from rats fed the BR931 diet with the same number order as lanes 5–8. For additional details see Materials and methods.

**Table II** Summary of abundance of various RNAs in rat liver tumours induced by BR931

| | Fold induction | |
|---|---|---|
| | *Tumour* | *'Normal'-adjacent* |
| Retrovirus-like sequences | | |
| RaLV | 1.98±0.58 | 1.32±0.54 |
| 30S | 0.97±0.29 | 0.80±0.19 |
| Proto-oncogenes | | |
| c-*myc* | 2.12±1.22 | 2.53±1.41 |
| c-H-*ras* | 0.87±0.24 | 1.02±0.18 |
| EGF receptor | 0.33±0.18 | 0.38±0.20 |
| ODC | 1.66±0.36 | 1.62±0.15 |

The data are expressed as described in Table I.

*c-H-*ras Messenger RNA transcripts, about 1.4 kilobases long, homologus to c-H-*ras* were detected in all of the liver samples from rats fed either the basal or BR931 diet. The results obtained with 12 representative samples are shown in Figure 1a. There was slight interindividual variation in the level of expression of the c-H-*ras* transcript between animals in the same treatment group. The BR931 diet led to a slight increase (about 2-fold) in the level of c-H-*ras* mRNA, when compared to age-matched rats fed the basal diet. The level of these transcripts was increased as early as day 3, but decreased to almost the basal level at day 28 (Table I).

The levels of expression of c-H-*ras* were not significantly different between liver tumours, the normal liver samples from age-matched controls and the 'normal' tumour-adjacent samples (Figure 2 and Table II).

*Epidermal growth factor (EGF) receptor* EGF receptor-related transcripts that were 10.5, 7.5 and 5.8 kilobases in size were found in all of the liver RNA samples obtained from rats fed either the basal or BR931 diet. The results obtained with 12 representative samples are shown in Figure 1c. There was some interindividual variation in the level of expression of EGF receptor transcripts between animals in the same treatment group. The BR931 diet caused a marked decrease (about 3.5-fold) in the level of EGF receptor RNA, when compared to age-matched rats fed the basal diet. The level of these transcripts was found to be decreased as early as day 3, and persisted throughout the experiment (Table I).

Messenger RNA transcripts homologous to EGF receptor were also detected in all of the liver tumour samples, the 'normal' tumour-adjacent tissues and the age-matched control samples (Figure 2). The relative abundance of these transcripts was decreased about 3-fold in the liver tumours induced by BR931, when compared to liver samples from the age-matched rats fed the basal diet (Table II). The relative abundance of these transcripts was, however, not significantly different between the 'normal' tumour-adjacent and liver tumour samples (Table II).

*Ornithine decarboxylase (ODC)* ODC-related transcripts that were 2.6 and 2.4 kilobases in size were seen in all of the liver RNA samples obtained from rats fed either the basal or BR931 diet. The results obtained with 12 representative samples are shown in Figure 1b. There was slight interindividual variation in the level of expression of the ODC transcript between animals in the same treatment group. The BR931 diet led to a slight increase (about 2-fold) in the level of ODC RNA, when compared to age-matched rats fed the basal diet (Table I). The level of these transcripts was found to be increased as early as day 3, and persisted throughout the experiment (Table I).

Messenger RNA transcripts homologous to ODC were also detected in the liver tumour samples, the 'normal' tumour-adjacent samples and the liver samples and the liver samples from normal age-matched controls (Figure 2). The relative abundance of these transcripts was increased (about 1.7-fold) in the liver tumours induced by BR931, when compared to the age-matched control samples (Table II). The relative abundance of these transcripts was, however, not significantly different between the 'normal' tumour-adjacent and liver tumour samples (Table II).

### Discussion

As mentioned in the *Introduction*, the mechanisms of hepatocarcinogenesis by BR931, a member of the peroxisome proliferator class of compound, are not well understood. The ability of this compound to induce peroxisome proliferation has been implicated in its carcinogenicity, presumably through the production of oxygen radicals by these organelles (Reddy *et al.*, 1980; Fahl *et al.*, 1984). However, recent studies indicate that the ability of these compounds to induce sustained enhancement of liver cell proliferation, and not the degree of peroxisome proliferation, correlates with the degree of tumour response (Marsman *et al.*, 1988). Furthermore, unlike hepatomas and their precursor lesions induced by classic hepatocarcinogens, those induced by hypolipidemic peroxisome proliferators do not express the enzymes γ-glutamyltranspeptidase and glutathione-S-transferase (Rao *et al.*, 1982; ibid, 1986). The present studies were, therefore, designed to investigate certain proliferation-related changes in gene expression in rats fed a BR931 containing diet for a 8 week period and also in liver tumours eventually produced by this diet.

Using Northern blot hybridisation analysis, we have found that the expression of endogenous RaLV-related sequences increased moderately during the first 7 days after the onset of the BR931 diet. Increased levels of these RNAs were also seen in liver tumours induced by the BR931 diet, when compared to normal liver samples from age-matched rats fed the basal diet. The levels of RNA transcripts homologous to 30S retrovirus-like sequences did not, however, change during the first 8 weeks of feeding the BR931 diet. Nor, was their increased expression of 30S-related RNAs in the liver tumours. Previously studies from this laboratory have shown that there is a marked increase in the expression of endogenous retroviral sequences related to both RaLV and 30S in diethylnitrosamine-induced rat hepatocellular adenomas and carcinomas (Hsieh *et al.*, 1987). We also found a dramatic increase in the level of RaLV-related RNAs but not 30S RNAs during liver regeneration induced by partial hepatectomy (Hsieh *et al.*, 1988). In cell culture studies we have also demonstrated increased expression of RaLV- and 30S- related sequences in log-phase rat fibroblast cells, when compared to quiescent cells (Hsieh & Weinstein, 1990). Thus increased expression of these endogenous retrovirus-related genes is often associated with cell proliferation, but the results obtained with the 30S sequence suggest that other factors also control its expression.

We observed a marked increases in the level of c-*myc* RNA during the first 8 weeks of feeding BR931. Higher levels of c-*myc* RNA were found in the control rat livers at day 3 than at day 56, which is consistent with the association of c-*myc* RNA with proliferation. After adjustment of this age factor, increased expression of c-*myc* (about 3-fold) was found throughout the 8 week period of feeding BR931. Increased expression of c-*myc* RNA was also observed in the liver tumour samples induced by BR931 diet, when compared to age-matched rats fed the basal diet. Previous results indicated that during rat liver regeneration there is a marked increase in the level of c-*myc* RNA, which precedes the peak of DNA synthesis (Hsieh *et al.*, 1988), suggesting that c-*myc* plays a role in regulating the entry of hepatocytes into the cell cycle. We observed only a slight increase in the level of c-H-*ras* RNA during the first 8 weeks of feeding the BR931 diet and no significant increase was seen in the liver tumour samples. A slight increase in the level of c-H-*ras* RNA was seen in regeneration rat liver (Hsieh *et al.*, 1988). Thus, increased expression of the c-H-*ras* gene does not appear to play an important role in hepatocyte proliferation.

ODC is the first and rate-limiting enzyme in the biosynthesis of polyamines in mammalian cells, and increases in ODC enzyme activity are frequently associated with cell proliferation (Pegg & McCann, 1982). In the present studies we found increased levels of ODC RNA in rat livers during the first 8 weeks of feeding the BR931 diet and also in the liver tumour samples induced by this diet. These results provide further evidence that this drug induces the expression of markers associated with cell proliferation.

We observed about a 3-fold decrease in the abundance of EGF receptor RNA transcripts in rat livers during the first 8 weeks of feeding the BR931 diet, and a similar decrease was seen in the liver tumours induced by this diet. Previous studies showed a marked suppression in EGF receptor binding in liver samples within 3 days after rats were fed the same BR931 diet (Gupta *et al.*, 1988), and other investigators (Bartles *et al.*, 1990) have described a decrease in EGF receptor protein and certain other plasma membrane proteins in rat liver after the administration of peroxisome proliferators. These investigators did not, however, examine EGF receptor mRNA levels. It has also been reported that partial hepatectomy and phenobarbital treatment caused a decrease in EGF receptor binding (Earp & O'Keefe, 1981; Hwang *et al.*, 1986; Eckl *et al.*, 1988), and in the level of EGF receptor mRNA (Hsieh *et al.*, 1988). Rat liver tumours induced by diethylnitrosamine also display a decrease in EGF receptor binding (Carr *et al.*, 1986) and EGF receptor mRNA (Hsieh *et al.*, 1987). A recent study indicates that the feeding of a methy-deficient diet also leads to a decrease in the level of EGF receptor mRNA in the livers of mice, within 7 days of the onset of this diet (Hsieh *et al.*, 1989). Taken together these findings provide strong evidence that alterations in the function of EGF may play an important role in liver cell proliferation and hepatocarcinogenesis, although the underlying mechanisms are not known at the present time.

In summary, the present studies indicate that the feeding of drug BR931, which induces a peroxisome proliferation in rodent livers and is a hepatocarcinogen, induces increased in the expression of several genes related to cell proliferation, including: RaLV-related sequences, c-*myc*, c-H-*ras*, and ODC; but decreased expression of the EGF receptor gene. Furthermore, liver tumours induced by the long term feeding of the BR931 diet showed similar changes in the expression of these genes. It is of interest that the increased levels of RNA for RaLV, 30S, c-*myc*, and ODC; and the decreased levels of RNA for EGF receptor seen in the liver tumours induced by BR931 were also seen in the 'normal' tumour-adjacent liver samples obtained from the same animals (Tables I and II). This suggests that the compound BR931 produces long term alterations in gene expression related to cell proliferation throughout the liver of rats receiving this compound. Further studies are required to determine whether the altered levels of specific mRNAs found in the present study reflect changes at the level of transcription or RNA stabilisation, and whether these changes are due to a primary effect of the drug BR931 or are secondary to the induction of peroxisome proliferation. Since these changes in gene expression occur relatively early (within 3 days) after onset of the feeding of this drug they may provide a useful marker for the mechanisms by which this and related drugs eventually induce liver tumours in rodents. Our findings on altered gene expression may also be relevant to the mechanisms of action of the peroxisome proliferator class of compounds, in view of the recent cloning of a peroxisome proliferator-activated receptor which is a member of the steroid hormone receptor superfamily. Presumably this protein functions as a transcription factor that is specifically activated by peroxisome proliferators and may, therefore, mediate the numerous effects of this class of agents (Isseman & Green, 1990).

This study was supported by National Cancer Institute Grants CA021111 (to I.B.W.), and CA26556 and CA40062 (to H.S.), the National Foundation for Cancer Research (to I.B.W.), and the American Institute of Cancer Research (to I.B.W.).

### References

AVIV, H. & LEDER, P. (1972). Purification of biologically active globin messenger RNA by chromatography onoligothymidylic acid-cellulose. *Proc. Natl Acad. Sci. USA*, **69**, 1408.

BARTLES, J.R., KHUON, S., LIN, X. & 6 others (1990). Peroxisome proliferator-induced alterations in the expression and modification of rat hepatocyte plasma membrane proteins. *Cancer Res.*, **50**, 669.

BISHOP, J.M. (1987). The molecular genetics of cancer. *Science*, **235**, 305.

BUTTERWORTH, B.E., LOURY, D.J., SMITH-OLIVER, T. & CATTLEY, R.C. (1987). The potential role of chemically induced hyperplasia in the carcinogenic activity of the hypolipidemic carcinogens. *Toxicol. Ind. Health*, **3**, 129.

CARR, B.I., ROITMAN, A., HWANG, D.L., BARSEGHIAN, G. & LEV-RAN, A. (1986). Effects of diethylnitrosamine on hepatic receptor binding and autophosphorylation of epidermal growth factor and insulin in rats. *J. Natl Cancer Inst.*, **77**, 219.

CHIRGWIN, J.M., PRZBYLA, A.E., MACDONALD, R.J. & RUTTER, W.J. (1979). Isolation of biologically active ribonucleic acid from sources enriched in ribonuclease. *Biochemistry*, **18**, 5294.

EARP, H.S. & O'KEEF, E.J. (1981). Epidermal growth factor receptor number decreases during rat liver generation. *J. Clin. Invest.*, **67**, 1580.

ECKL, P.M., MEYER, S.A., RIVE'WHITCOMBE, W. & JIRTLE, R.L. (1988). Phenobarbital reduces EGF receptors and the ability of physiological concentrations of calcium to suppress hepatocytes proliferation. *Carcinogenesis*, **9**, 479.

ELLIOTT, B.M. & ELCOMBE, C.R. (1987). Lack of DNA damage or lipid peroxidation measured *in vivo* in the rat liver following treatment with peroxisomal proliferators. *Carcinogenesis*, **8**, 1213.

ELLIS, R.W., DE FEO, D., MARYAK, J.M. & 5 others (1980). Dual evolutionary origin for the rat genetic sequences of Harvey murine sarcoma virus. *J. Virol.*, **36**, 408.

FAHL, W.E., LALWANI, N.D., WATANABE, T., GOEL, S.K. & REDDY, J.K. (1984). DNA damage related to increased hydrogen peroxide generation by hypolipidemic drug-induced liver peroxisomes. *Proc. Natl Acad. Sci. USA*, **81**, 7827.

GONDA, M.S., YOUNG, H.A., ELSER, J.E. & 5 others (1982). Molecular cloning genomic analysis, and biological properties of rat leukemia virus and the *onc* sequences of Rasheed rat sarcoma virus. *J. Virol.*, **44**, 520.

GOVINDARAJULU, Z. (1988). *Statistical Techniques in Bioassay*. S Karger AG: Basel.

GUILLEM, J.G., HSIEH, L.L., O'TOOLE, K.M., FORDE, K.S., LOGERFO, P. & WEINSTEIN, I.B. (1988). Changes in expression of oncogenes and endogenous retroviral-like sequences during colon carcinogenesis. *Cancer Res.*, **48**, 3964.

GUPTA, C., HATTORI, A. & SHINOZUKA, H. (1988). Suppression of EGF binding in rat liver by the hypolipidemic perosisome proliferators, 4-ahloro-6-(2,3-xylidino)-2-pyrimidinylthio-(N-b-hydrozy ethyl)acetamide and di(2-ethylhexyl)phthalate. *Carcinogenesis*, **9**, 167.

HSIEH, L.L., HSIAO, W.-L., PERAINO, C., MARONPOT, R.R. & WEINSTEIN, I.B. (1987). Expression of retroviral sequences and oncogenes in rat liver tumors induced by diethylnitrosamine. *Cancer Res.*, **47**, 3421.

HSIEH, L.L., PERAINO, C. & WEINSTEIN, I.B. (1988). Expression of endogenous retrovirus-like sequences and cellular oncogenes during phenobarbital treatment and regeneration in rat liver. *Cancer Res.*, **48**, 265.

HSIEH, L.L., WAINFAN, E., HOSHINA, S., DIZIK, M. & WEINSTEIN, I.B. (1989). Altered expression of retrovirus-like sequences and cellular oncogenes in mice fed methyl-deficient diets. *Cancer Res.*, **49**, 3795.

HSIEH, L.L. & WEINSTEIN, I.B. (1990). Factors influencing the expression of endogenous retrovirus-like sequences in Rat 6 cells. *Mol. Carcinogenesis*, **3**, 344.

HWANG, D.L., ROITMAN, A., LEV-RAN, A. & CARR, B.I. (1986). Chronic treatment with phenobarbital decreases the expression of rat liver EGF and insulin receptors. *Biochem. Biophys. Res. Commun.*, **135**, 501.

ISSEMANN, I. & GREEN, S. (1990). Activation of a member of the steroid hormone receptor superfamily by peroxisome proliferators. *Nature*, **347**, 645.

KAHANA, C. & NATHANS, D. (1985). Nucleotide sequence of murine ornithine decarboxylase mRNA. *Proc. Natl Acad. Sci. USA*, **82**, 1673.

LAMBERT, M.E., GATTONI-CELLI, S., KIRSCHMEIER, P. & WEINSTEIN, I.B (1983). Benzo[a]pyrene induction of extrachromosomal viral DNA synthesis in rat cells transformed by polyoma virus. *Carcinogenesis*, **4**, 587.

MARSMAN, D.S., CATTLEY, R.C., CONWAY, J.G. & POPP, J.A. (1988). Relationship of hepatic peroxisome proliferation and replicative synthesis to the hepatocarcinogenicity of the peroxisome proliferators di(2-ethylhexyl)phthalate and [4-chloro-6-(2,3-xylidino)-2-pyrimidinyl-thio]acetic acid (Wy-14, 643) in rats. *Cancer Research*, **48**, 6739.

PEGG, A.E. & MCCANN, P.P. (1982). Polyamine metabolism and function. *Am. J. Physiol.*, **243**, C212.

POPP, J.A., GARVEY, L.K. & CATTLEY, R.C. (1987). *In vivo* studies on the mechanism of di(2-ethylhexyl)phthalate carcinogenesis. *Toxicol. Ind. Health*, **3**, 151.

RANDERATH, E., RANDERATH, K., REDDY, R., RAO, M.S. & REDDY, J.K. (1989). Rat liver DNA alterations induced by the peroxisome proliferator ciprofibrate. *Proc. Am. Assoc. Cancer Res.*, **30**, 146.

RAO, M., LALWANI, N.D., SCARPELLI, D.G. & REDDY, J.K. (1982). The absence of γ-glutamyltranspeptidase activity in putative preneoplastic lesions and in hepatocellular carcinomas induced in rats by the hypolipidemic peroxisome proliferator Wy-14, 643, *Carcinogenesis*, **3**, 1231.

RAO, M.S., TATEMATSU, M., SUBBARAO, V., ITO, N. & REDDY, J.K. (1986). Analysis of peroxisomal proliferator-induced preneoplastic and neoplastic lesions of rat liver for placental form of glutathione-S-transferase and γ-glutamyltranspeptidase. *Cancer Res.*, **46**, 5287.

RAO, M.S. & REDDY, J.K. (1987). Peroxisome proliferation and hepatocarcinogenesis. *Carcinogenesis*, **8**, 631.

REDDY, J.K., AZARNOFF, D.L. & SIRTORI, C.R. (1987). Hepatic peroxisome proliferation: induction by BR931, a hypolipidemic analog of WY-14, 643. *Arch. Int. Pharmacodyn.*, **234**, 4.

REDDY, J.D., AZARNOFF, D.L. & HIGNITE, D.D. (1980). Hypolipidemic hepatic peroxisome proliferators from a novel class of chemical carcinogens. *Nature*, **283**, 397.

REDDY, J.K. & LALWANI, N.D. (1983). Carcinogenesis by hepatic peroxisome proliferators: evaluation of the risk of hypolipidemic drugs and industrial plasticizers to human. *CRC Crit. Rev. Toxicol.*, **12**, 1.

REDDY, J.K. & RAO, M.S. (1986). Peroxisome proliferators and cancer: mechanisms and implications. *Trends Pharmacol. Sci.*, **7**, 438.

RIGBY, P.W.J., DIECKMANN, R., RHODES, C. & BERG, P. (1977). Labeling of deoxyribonucleic acid to high specific activity *in vitro* by nick translation with DNA polymerase. *I.J. Mol. Biol.*, **113**, 237.

RONAI, A.A., OKIN, E. & WEINSTEIN, I.B. (1988). Ultraviolet light induces the expression of oncogenes in rat fibroblast and human keratinocyte cells. *Oncogene*, **2**, 201.

SIRTORI, C.R., CATAPANO, A. & PAOLETTI, R. (1977). Therapeutic significance of hypolipidemic and antiatherosclerotic drugs. *Atherosclerosis Rev.*, **2**, 113.

STANTON, L.W., WATT, R. & MARCU, K.B. (1983). Translocation, breakage and truncated transcripts of c-*myc* oncogene in murine plasmacytomas. *Nature*, **303**, 401.

ULLRICH, A., COUSSENS, L., HAYFLICK, J.S. & 12 others (1984). Human epidermal growth factor cDNA sequence and aberrant expression of the amplified gene in A431 epidermoid carcinoma cells. *Nature*, **309**, 418.
WAHL, G.M., STERN, M. & STARK, G.R. (1979). Efficient transfer of large DNA fragments from agarose gels to diazobenzylozymethl-paper and rapid hybridization by using dextran sulfate. *Proc. Natl Acad. Sci. USA*, **76**, 3683.
WEINBERG, R.A. (1989). Oncogenes, antioncogenes, and the molecular bases of multistep carcinogenesis. *Cancer Res.*, **49**, 3713.
YOUNG, H.A., GONDA, M.A., DE FEO, D. & SCOLNICK, E.M. (1980). Heteroduplex analysis of cloned rat endogenous replicative-defective (30S) retrovirus and Harvey murine sarcoma virus. *Virology*, **107**, 89.